# Supplementary material for: Raman Spectroscopy and Thermoelectric Characterization of Composite Thin Films of Cu2ZnSnS4 Nanocrystals Embedded in a Conductive Polymer PEDOT:PSS
Source: Nanomaterials (Basel). 2022 Dec 22;13(1):41. doi: 10.3390/nano13010041 (PMC9824269; doi:10.3390/nano13010041)
Supplement: Supplementary file 1 [file nanomaterials-13-00041-s001.zip › FLA treated CZTS NCs - PEDOT PSS_SI.pdf]

Supplementary information

Raman spectroscopy and thermoelectric characterization of composite thin films of Cu_2ZnSnS_4 nanocrystals embedded in a conductive polymer PEDOT:PSS

Ye. Havryliuk[1,2,3,*], V. Dzhagan[3,4], A. Karnaukhov[3], O. Selyshchev[1,2], J. Hann[2], D.R.T. Zahn[1,2]

[1] Semiconductor Physics, Chemnitz University of Technology, 09107, Chemnitz, Germany
[2] Center for Materials, Architectures, and Integration of Nanomembranes (MAIN), 09126 Chemnitz, Germany
[3] V.E. Lashkaryov Institute of Semiconductor Physics NAS of Ukraine, 03028 Kyiv, Ukraine
[4] Physics Department, Taras Shevchenko National University of Kyiv, 60 Volodymyrs'ka str., 01601 Kyiv, Ukraine
* Correspondence: yevhenii.havryliuk@physik.tu-chemnitz.de

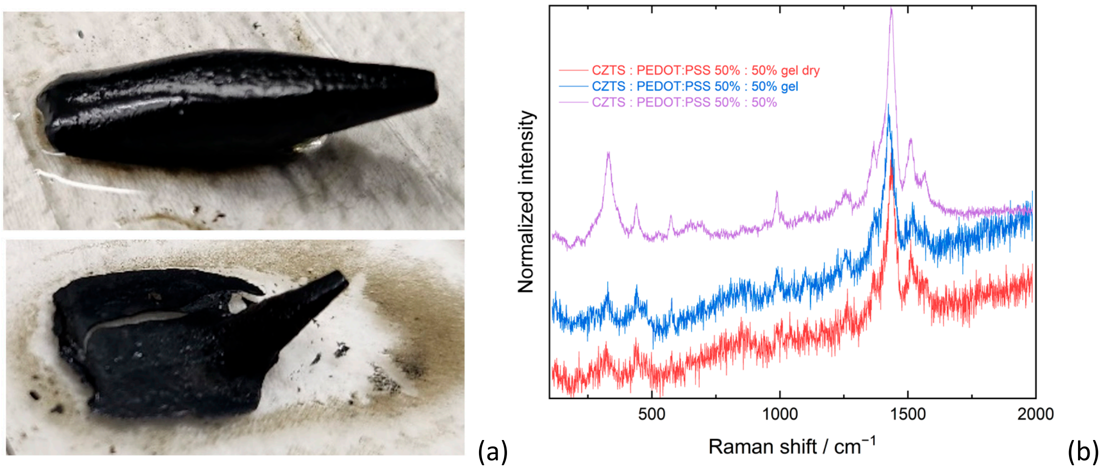

Figure S1. (a) Fresh gel (upper photo) and after drying under the hood for 12 hours (bottom). (b) Raman spectra of fresh mixture, fresh gel, and gel after drying.

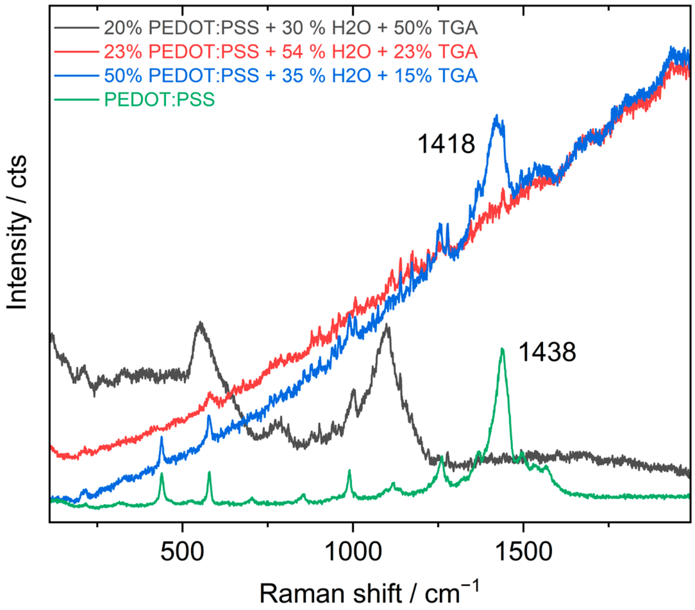

Figure S2. Control experiment with effect of pure TGA solution on the Raman spectrum of PEDOT:PSS.

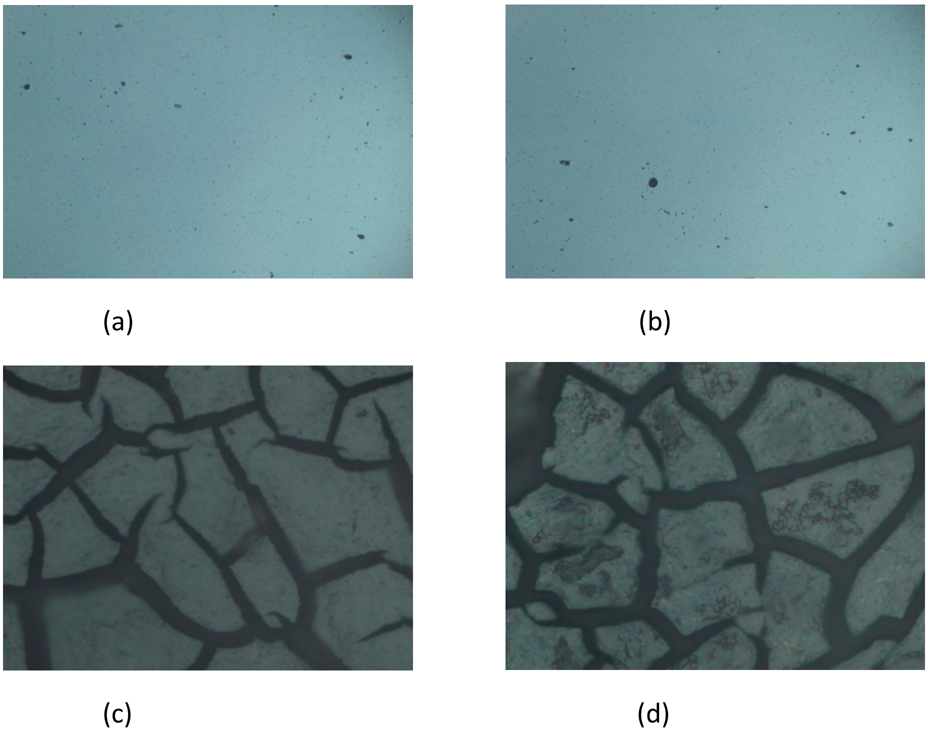

Figure S3. Optical image of PEDOT:PSS film (a) before and (b) after 12J/cm^2 FLA; and CZTS NCs film (c) before and (d) after 12J/cm^2 FLA
