# Supplementary figures and images for: Raman Spectroscopy and Thermoelectric Characterization of Composite Thin Films of Cu2ZnSnS4 Nanocrystals Embedded in a Conductive Polymer PEDOT:PSS
Source: Nanomaterials (Basel). 2022 Dec 22;13(1):41. doi: 10.3390/nano13010041 (PMC9824269; doi:10.3390/nano13010041)

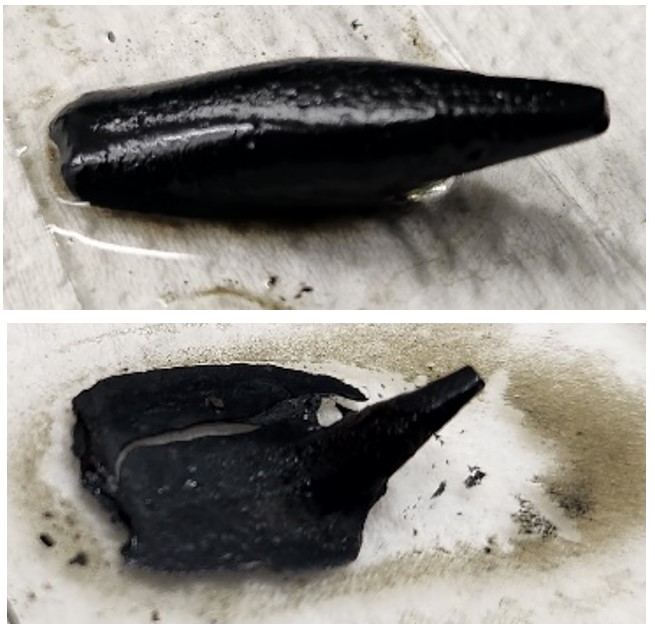

Supplement: Supplementary file 1 [file nanomaterials-13-00041-s001.zip › S1a.jpg]

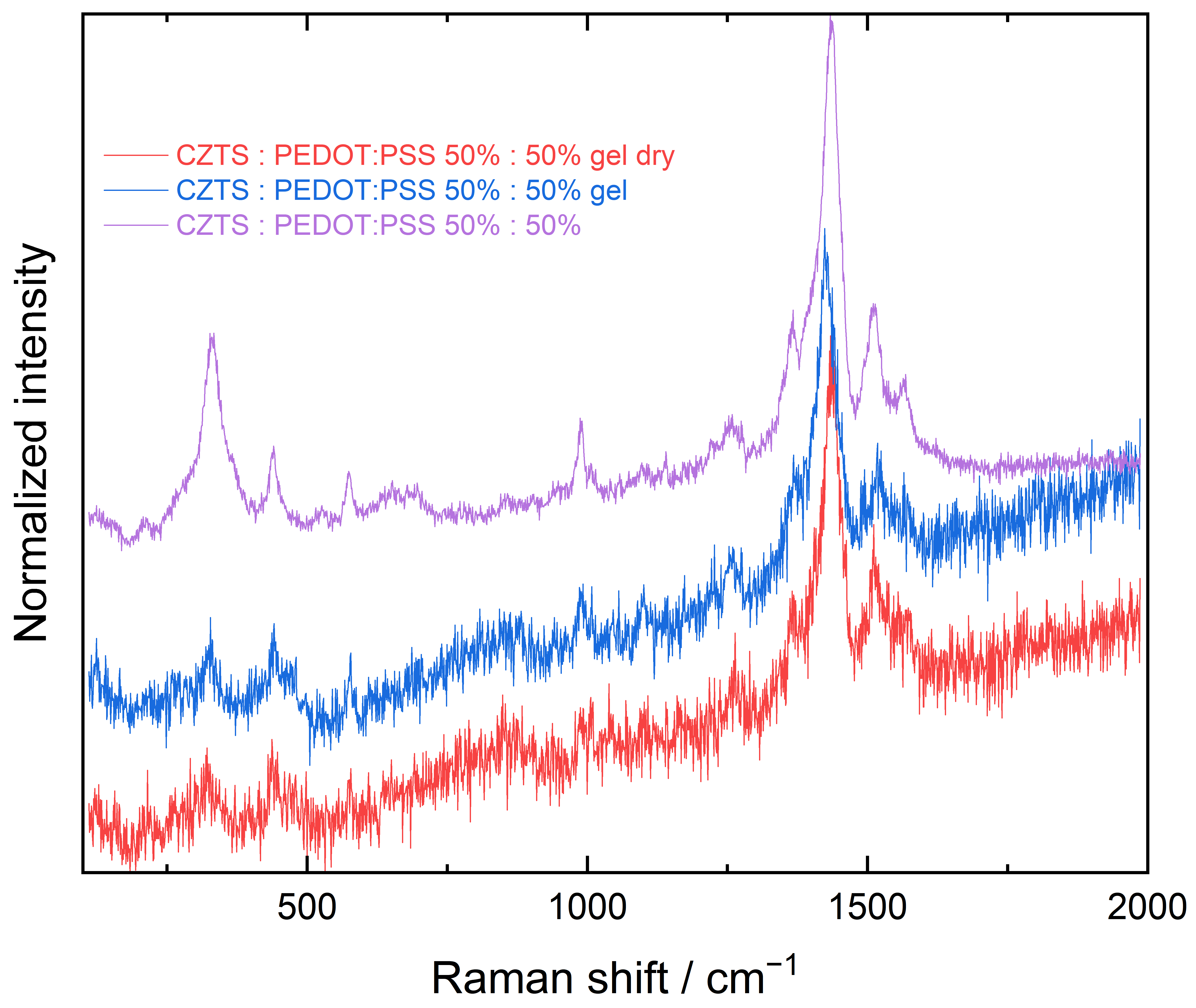

Supplement: Supplementary file 1 [file nanomaterials-13-00041-s001.zip › S1b.gif]

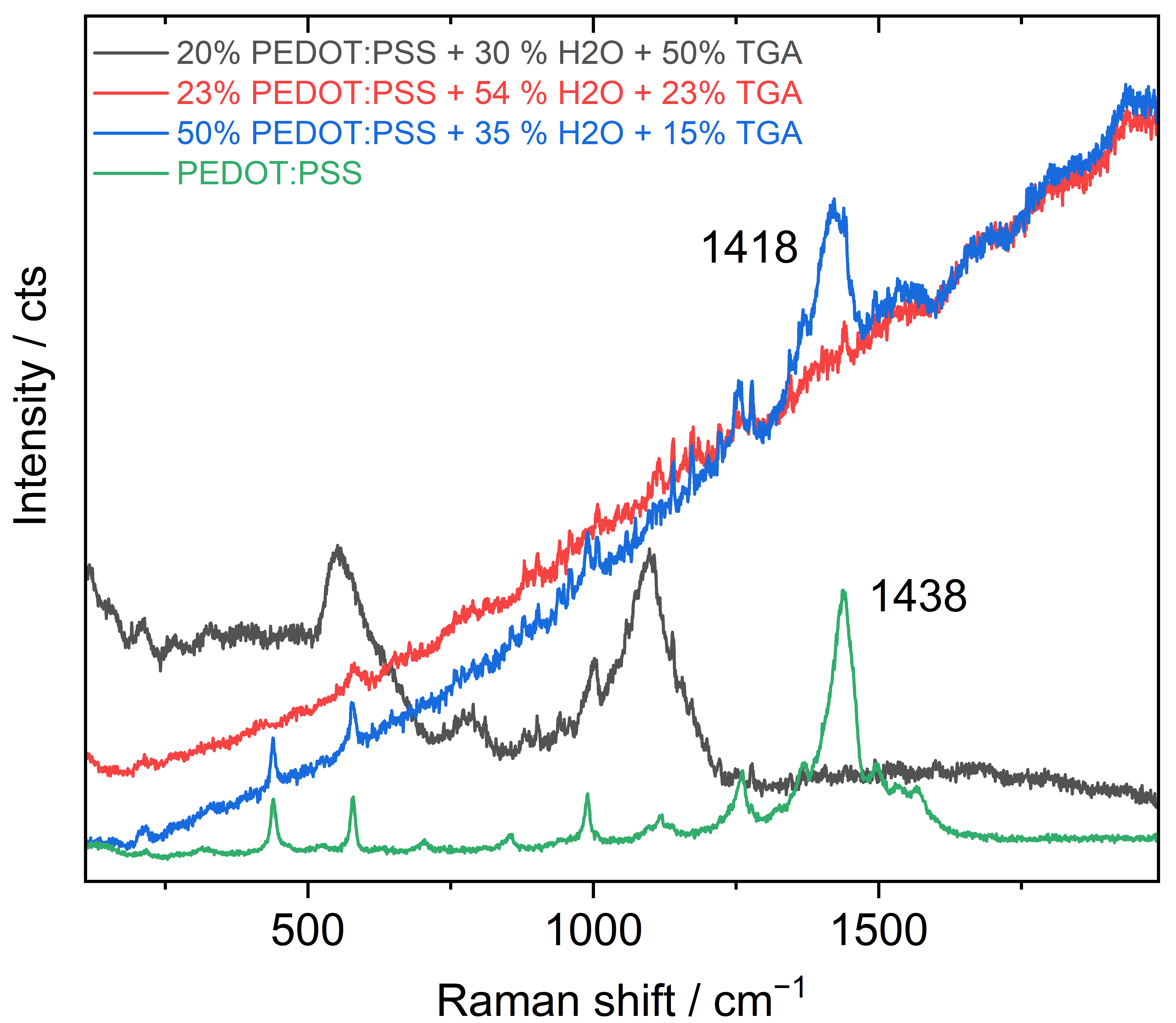

Supplement: Supplementary file 1 [file nanomaterials-13-00041-s001.zip › S2.gif]

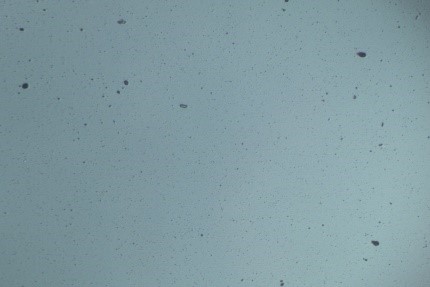

Supplement: Supplementary file 1 [file nanomaterials-13-00041-s001.zip › S3a.jpg]

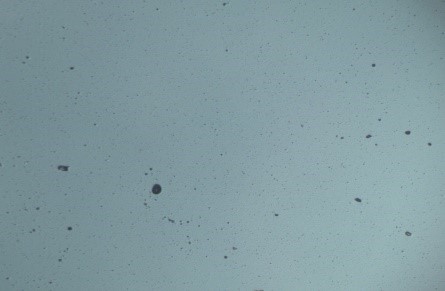

Supplement: Supplementary file 1 [file nanomaterials-13-00041-s001.zip › S3b.jpg]

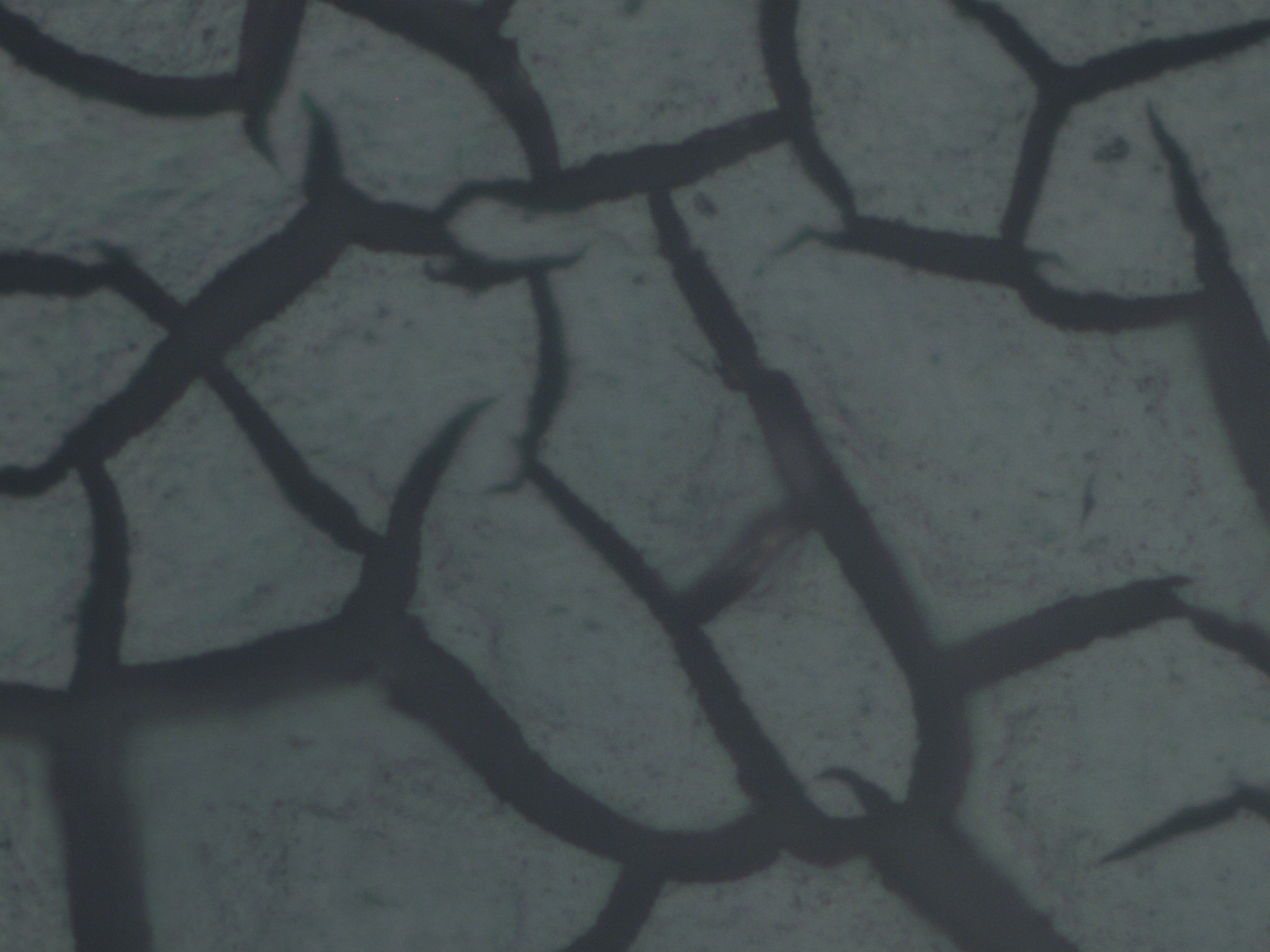

Supplement: Supplementary file 1 [file nanomaterials-13-00041-s001.zip › S3c.jpg]

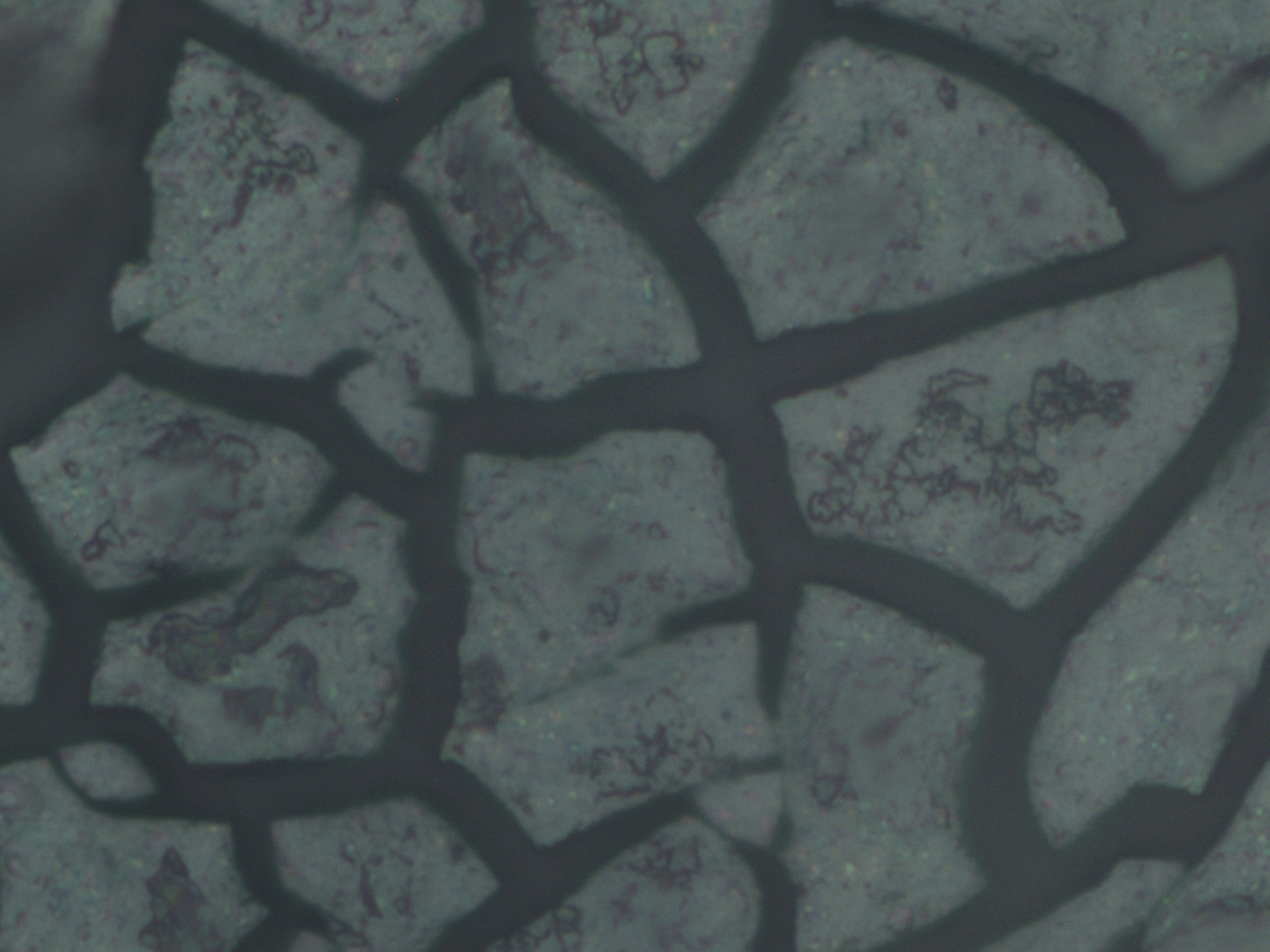

Supplement: Supplementary file 1 [file nanomaterials-13-00041-s001.zip › S3d.jpg]
